# Humoral and cell-mediated immunity to MSP3 peptides in adults immunized with MSP3 in malaria endemic area, Burkina Faso

**DOI:** 10.1111/j.1365-3024.2009.01130.x

**Published:** 2009-08

**Authors:** I NEBIE, A DIARRA, A OUEDRAOGO, A B TIONO, A T KONATE, A GANSANE, I SOULAMA, S COUSENS, O LEROY, S B SIRIMA

**Affiliations:** 1 Centre National de Recherche et de Formation sur le PaludismeOuagadougou, Burkina Faso; 2 London School of Hygiene and Tropical MedicineLondon, UK; 3 European Malaria Vaccine Initiative (EMVI), Centre for International Health BergenBergen, Norway

**Keywords:** adults, immunogenicity, merozoite surface protein-3, phase 1b

## Abstract

We performed a single-blind, randomized phase 1 trial of the long synthetic peptide (LSP) of merozoite surface protein-3 (MSP3) in adults living in Burkina Faso. Thirty eligible volunteers were randomized to receive either the MSP3-LSP candidate vaccine or tetanus toxoid vaccine as a control. A dose of each vaccine was administered on days 0, 28 and 112 and the vaccine was formulated with aluminium hydroxide. Humoral immune responses were assessed by ELISA at days 0, 28, 56, 112, 140, 252 and 365 and cell-mediated immune responses by lymphoproliferation assay and by ELISA on days 0, 56 and 140. IgG responses to four peptides of MSP3 were similar in both vaccine groups. Higher IgG concentrations were recorded after the beginning of malaria high transmission season in both vaccine groups. The lymphocyte proliferation and the production of IFN-γ in response to stimulation with the four overlapping peptides increased following vaccination in the MSP3-LSP vaccine group, but did not change appreciably in the control group. In contrast to natural infection, MSP3-LSP did not boost humoral responses to the four overlapping peptides of MSP3 to any detectable degree in our semi-immune adult. MSP3-LSP may be more immunogenic in young children with little or no acquired immunity.

## Introduction

An effective antimalarial vaccine remains a highly desirable goal. Several *Plasmodium falciparum* antigens identified as vaccine candidates have been sequenced and immunologically characterized. Among these antigens, merozoite surface protein-3 (MSP3) of the asexual stage has emerged as a promising candidate (1–3). In contrast to the N-terminus, the C-terminal part of the molecule, is highly conserved among field isolates of the parasite (4,5).

Many seroepidemiological and laboratory studies conducted with samples from different settings have reported that cytophilic antibodies to MSP3 (IgG1 and IgG3) predominate in protected individuals while unprotected individuals produce mostly noncytophilic antibodies (IgG2 and Ig4) (1,2). Three peptides (b, c and d) from the C-terminal region of MSP3 were used to affinity purify antibodies that possessed antibody-dependant cellular inhibition activity from sera from a population in which malaria is endemic (6). The transfer of human monocytes together with antibodies to MSP3 induced rapid clearance of parasites in an immunocompromised mouse model of *P. falciparum* (7). In *Saimiri sciureus* and *Aotus nancymai*, recommended as primate models for malaria research by WHO, MSP3 can induce protective immunity against experimental *P. falciparum* infection (7–10). Two human phase 1 MSP3 vaccine trials have been conducted in malaria-naïve and in semi-immune adults (11,12). In malaria-naïve, Swiss adults MSP3 long synthetic peptide (MSP3-LSP) was reported to be safe and to induce a marked specific anti-MSP3-LSP antibody response, an anti-native MSP3 antibody response, a T-cell antigen-specific proliferative response and gamma interferon production (11). In semi-immune males aged 18–40 years, living in a malaria-endemic area MSP3-LSP was reported to be safe and immunogenic (12).

In this paper, we present in more detail the immunological responses (IgG antibodies, lymphocyte proliferation and gamma interferon production) to four overlapping peptides of MSP3 of the semi-immune participants in the latter trial in Burkina Faso. The four overlapping small peptides MSP3-a, MSP3-b, MSP3-c and MSP3-d, which span the MSP3-LSP sequence, each define a B-cell epitope and a T-cell epitope (6,11). Antibody responses to peptides b, c and d have been associated with protection in the past (6,13).

## Materials and methods

### Study site

The study site is described elsewhere (12). Briefly, the study was conducted at the Medical Centre, Sector 30, Ouagadougou in Burkina Faso. Malaria transmission is seasonal, being low during the dry season (November to May) and high during the rainy season from June to October. During the rainy season, the estimated incidence of clinical malaria in children under 5 years of age is one episode per child with *P. falciparum* accounting for more than 95% of infections.

### Study participants

Thirty male volunteers, aged 18–40 years living in the village of Balonghin, a village 50 km south of Ouagadougou, were recruited under a protocol approved by the ethical committee for medical research of the Ministry of Health, Burkina Faso and by the Ethics Committee of the London School of Hygiene and Tropical Medicine. The trial was conducted in compliance with the International Conference on Harmonization’s Good Clinical Practice principles, the Declaration of Helsinki and the regulatory requirements of Burkina Faso. Individual written informed consent was obtained from all participants. Individuals were eligible for inclusion in the trial if they were found to be healthy at a general medical examination, indicated their intention to reside in the village for the duration of the trial (12 months) and gave written informed consent. Exclusion criteria included: (i) symptoms of any condition that could interfere with the interpretation of the trial results or compromise the health of the subject; (ii) any clinically significant, abnormal haematological parameters; (iii) seropositivity to HIV, HBV or HCV; (iv) *Schistosoma haematobium* infection; (v) history of immunosuppressive therapy (steroids, immunomodulator or immunosuppressor) within the 3 months preceding enrolment; (vi) suspected or known hypersensitivity to any of the vaccine components or to a previously administered vaccine and (vii) history of complete anti-tetanus vaccination. Each participant was assigned a unique identification number and given an identity card to assist correct identification subsequently.

### Study design

The study was designed as a single-blind, randomized trial to assess the safety and the immunogenicity of three doses of 30 μg of MSP3-LSP adsorbed on aluminium hydroxide (12). Thirty eligible volunteers (15 per arm) were randomized to receive either the MSP3-LSP candidate vaccine or tetanus toxoid (TT) vaccine as a control. A dose of each vaccine was administered on days 0, 28 and 112. The volunteers were transported from Balonghin to the Medical Centre for vaccination and for planned follow-up visits. The laboratory staff who performed the immunological assays were not aware which study participants had been allocated to each arm.

### Vaccines

The MSP3-LSP vaccine is an LSP containing the amino-acid sequence 186–276 of the *P. falciparum* MSP-3. The vaccine was manufactured by Dictagene (Epalinges, Switzerland) under good manufacturing practices conditions, produced in lyophilized form and delivered in multidose vials; excipients were sodium chloride 9 mg/mL, trisodium-citrate 10 mm (2·94 mg/mL), disodium-phosphate buffer 10 mm (1·42 mg/mL). Before administration, the vaccine was reconstituted using 1 mL of isotonic saline solution (9‰) and mixed with 1 mL of aluminium hydroxide, under sterile conditions (under a laminar hood), following the procedure recommended by the manufacturer. A minimum interval of 60 min was allowed between reconstitution and administration of the vaccine to allow adequate adsorption of the vaccine. The adsorbed vaccine was aliquoted into single-use syringes (BioCare Nordic ApS, Roskilde, Denmark). From each multidose vial, three individual vaccine doses of 0·5 mL, containing 30 μg of peptide, were prepared.

The TT vaccine used was produced from a formaldehyde detoxified and purified tetanus toxin provided by the Statens Serum Institute, Copenhagen, Denmark (batch no. 7906). The vaccine was reconstituted using the same adjuvant as the MSP3-LSP vaccine (aluminium hydroxide and buffer solution).

### Determination of immunological parameters

#### Blood sampling

Blood for the analysis of both humoral and cellular immune responses was obtained before each immunization and at specific time points during the study.

Humoral immune responses on days 0, 28, 56, 112, 140, 252 and 365 were evaluated using whole blood samples collected on heparin as an anticoagulant (VF-109SHL, Terumo Europe n.v, Leuven, Belgium). Lymphocyte proliferation and cytokine production were only measured on days 0, 56 (28 days after the second injection at day 28) and 140 (28 days after the last injection at day 112).

The first blood draw for immunological assays (on day 0) was done in October 2003 (1 month before the end of malaria high transmission period) and the last (day 252) was done at the end of June 2004, shortly after the beginning of the rainy season at a time when malaria transmission was beginning to increase after the low levels of the dry season.

#### MSP3-LSP and MSP3 overlapping peptides

We studied immunological responses using the following MSP3 peptides:

1. The vaccine peptide MSP3-LSP: this corresponds to a fully conserved region covering amino acids 181–276 (product number 00FS021#1B, Dictagene) (sequence RKTKEYAEKAKNAYEKAKNAYQKANQAVLK AKEASSYDYILGWEFGGGVPEHKKEENMLSHLYVSSKDKENISKENDDVLDEKEEEAEETEEEELE) of the C-terminal region of MSP3 (386 amino acids long) from the *P. falciparum* strain Fc27 (4).

2. Four overlapping peptides (a, b, c and d) which span 86% of MSP3-LSP: MSP3-a amino acids 194–217 (Product number Dicta-F64#1C, Dictagene) (HERAKNAYQKANQAVLKAKEASSY); MSP3-b amnino acids 211–237 (Product number Dicta-G40#1C, Dictagene) (AKEASSYDYILGWEFGGGVPEHKKEEN); MSP3-c amino acids 230–257 (Product number Dicta-G65#1C, Dictagene SA) (PEHKKEENMLSHLYVSSKDKENISKENE) and MSP3-d amino acids 238–276 (Product number Dicta-G41#1C, Dictagene) (MLSHLYVSSKDKENISKENDDVLDEKEEEAEETEEEELE).

These peptides were synthesized, purified, bottled and lyophilized following GMP procedures (batch no. 00FS023, RMF Dictagene).

Tetanus toxoid (ATP PTC 10005 PREP No 2, Pasteur Merieux, Marcy L’Etoile, France) and phytohemagglutinin (PHA) (PHA-L, cat# L2769; Sigma) were used as controls.

#### Assessment of antibody responses

Merozoite surface protein-3 long synthetic peptide-specific IgG concentrations were measured by ELISA. Concentrations of IgG specific to the four overlapping peptides of MSP3 (a, b, c and d) were also measured. The ELISA was done according to the Afro Immuno Assay standard operating procedure (SOP number AIA-007-03) (3,12). In brief, microtitre plates (NUNC – Maxisorp F 96 439454, Roskilde, Denmark) were coated with the appropriate synthetic peptide (1 μg/mL), incubated overnight at 4°C, and blocked with 3% milk powder (cat# 92964, Marvel, Dublin, Ireland) in PBS-Tween 20 for 1 h. Plasma samples diluted 1 : 200 were added in duplicate and incubated at room temperature for 2 h. Plates were washed four times between each step. Plates were developed with either peroxidase conjugated goat anti-human IgG (secondary antibody) (Caltag– H10007, Carlsbad, California, USA). The revealing was done with peroxidase conjugated goat anti-mouse IgG.

Bound secondary antibody for IgG were quantified by colouring with ready to use TMB (3,3′,5,5′-tetramethylbenzidine) substrate (cat# 4390A; Kem-En-Tec diagnostics, Taastrup, Denmark). Optical density (OD) was read at 450 nm with a reference at 620 nm, and the OD value of the test-sample were converted into arbitrary units by means of a standard curve on each plate.

For antibody quantification, to control for interassay and day-to-day variations in the standardized ELISA procedure, threefold serial dilutions of reference standard reagents (IgG) were directly coated on each ELISA plate at a starting concentration of 1000 ng/mL (100 μL/well). Optical density values for the test samples were converted into antibody units with the standard reference curves generated for each ELISA plate using a four-parameter curve-fit microsoft excel-based application. Samples were re-tested if the coefficient of variation between duplicate absorbance values were higher than 15% and plates were also re-tested if the *R*-square value of the standard curve was less than 97%.

However if the OD at 1 : 200 was above the readable range of the ELISA plate reader, the test sample was further diluted.

The positive control plasmas were from positive Liberian plasma samples and the negative controls from Danish plasma samples graciously provided by Michael Theisen from Statens Serum Institute (Copenhagen, Denmark). Plates not meeting pre-defined limits for control values were excluded and the samples re-tested.

#### Assessment of cellular immune responses

Peripheral blood mononuclear cells (PBMC) were isolated from heparinized venous whole blood by gradient centrifugation on Ficoll-Hypaque (Histopaque 1077; Sigma® 1077-1). For the proliferative assays, PBMC were washed and adjusted to a concentration of 2 × 10^6^ cells/mL in Dulbecco’s MEM supplemented with HEPES buffer, 100-U/mL penicillin/streptomycin, nonessential amino acids and heat-inactivated human AB serum. Fresh PBMC were distributed in sextuplicates in 96-well flat-bottom plates (2 × 10^5^ cells/well) (Costar cat# 3596; Corning Incorporated, New York, USA). Peripheral blood mononuclear cells were stimulated with a range of concentrations of MSP3-a, MSP3-b, MSP3-c and MSP3-d peptides (2 and 10 μg/mL), with TT (10 μg/mL) and PHA (10 μg/mL) as positive controls and incubated at 37°C in 5% CO_2_/95% air. Peripheral blood mononuclear cells from each volunteer were incubated in the same conditions in media alone to serve as negative control. After 6 days, 100-μL supernatant was removed and replaced with proliferation medium containing [^3^H]-thymidine (1 μCi/well). The cells were harvested 24 h later onto glass fibre filters and [^3^H]-thymidine incorporation was measured by liquid scintillation (Wallac scintillation products, Milton Keynes, UK) in a β-counter. The stimulation index (SI) was defined as the mean count per minute of experimental wells divided by the mean of negative control wells (cells and medium alone).

Production of IFN-γ in supernatants of PBMC prepared and plated as described for proliferation assay, and stimulated with 10 μg/mL for MSP3 fragments or controls, was measured by ELISA (Elipair, Diaclone, Besançon, France), following the manufacturer’s instructions.

### Data analysis

The database was created in microsoft excel. Descriptive analysis of the data were undertaken using stata (version 8·2; College Station, TX, USA). Given the small sample size, formal statistical analysis were not used to compare vaccine groups. Median concentrations of IgG, SI and IFN-γ concentration and 95% confidence interval were calculated by using log_10_-transformed values.

## Results

### Humoral responses to MSP3-LSP and to four overlapping peptides

**Figure 1:**
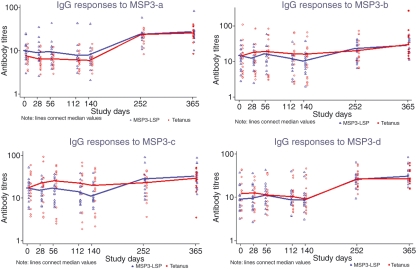
Total IgG to MSP3 overlapping peptides at different timepoints, by vaccine group.

To identify immunogenic regions, IgG responses to four overlapping peptides MSP3-a, MSP3-b, MSP3-c and MSP3-d were examined. No major differences in responses between the two vaccine groups were observed (Figure 1). Higher IgG concentrations against the peptides were recorded after day 140 at the beginning (day 252) and peak of malaria high transmission season (day 365) in both vaccine groups. The highest antibody responses were generally to MSP3-c.

### Cell-mediated immunity to MPS3-LSP and to four overlapping peptides

**Figure 2:**
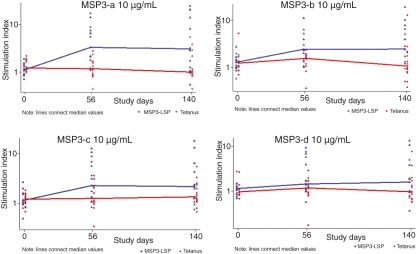
Lymphocyte stimulation index in the presence of MSP3 fragments at different timepoints, by vaccine group.

Lymphocyte proliferation in response to MSP3 fragments is shown in Figure 2. Two different concentrations (10 and 2 μg/mL) of the four overlapping peptides were used to stimulate the PBMC (data for 2 μg/mL are not shown). The stimulation was higher with the 10 μg/mL. The SIs were similar in both vaccine groups at day 0 but increased in MSP3-LSP vaccine group post-vaccination (days 56 and 140) for all fragments, with the strongest response being to peptide MSP3-a (Figure 2). At the higher concentration of 10 μg/mL at day 56 73% (11/15), 73% (11/15), 53% (8/15) and 40% (6/15) of volunteers of MSP3-LSP vaccine group had SI at least the double of those observed at D0, respectively, for MSP3-a, MSP3-b, MSP3-c and MSP3-d. Between 0 and 27% of volunteers had similar responses in the control group.

**Figure 3:**
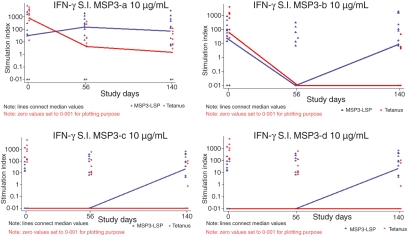
IFN-γ production in the presence of MSP3 fragments at different timepoints, by vaccine group.

IFN-γ was measured in PBMC supernatants following stimulation with the four MSP3 overlapping peptides at 10 μg/mL before the first vaccination and after the second and the third vaccinations (Figure 3). In the MSP3-LSP group, IFN-γ levels following stimulation with MSP3 fragments remained relatively stable over time while in the control arm IFN-γ levels declined substantially.

## Discussion

The aim of this paper was to investigate the pattern of humoral and cell-mediated immunity to the four overlapping peptides of the highly conserved C-terminal part of MSP3 in semi-immune adults from 20 to 45 years immunized with MSP3-LSP, a malaria vaccine candidate. Previous experience has shown that LSP were safe and immunogenic (14–19). Despite the small sample size, the results presented in this paper suggest that, in contrast to natural infection, MSP3-LSP vaccine adsorbed on aluminium hydroxide did not boost humoral responses to the four overlapping peptides of MSP3. The vaccine may, however, have boosted some components of cell-mediated immunity.

The findings with respect to humoral responses against the short peptides are similar to the those for IgG responses against MSP3-LSP obtained with the same volunteers (12) and are in contrast to the results of the phase 1 trial conducted in naïve Swiss adults who displayed strong, specific anti-MSP3-LSP antibody responses (11). IgG antibody concentrations to MSP3-LSP (12) and to MSP3 fragments in naturally exposed adults were already high at baseline, presumably as a result of exposure to natural infection and the vaccine did not appear to have any boosting effect. Previous trials conducted in Kenya and Papua New Guinea have reported similar findings (20,21). The authors observed no significant difference in antibody titres against some peptides between the controls and the vaccines groups. The most likely explanation is that the high intensity transmission may have overshadowed the inductive capacity of the vaccine. Antibody concentrations measured at the two time points after day 140, in June and October during the high transmission period, suggest a modest boosting effect of exposure to natural infection.

The magnitude of IgG responses to these peptides was highest for IgG against MSP3-c, whose antiparasitic effect has been already demonstrated (6). However, we have not been able to show that antibody patterns to each region differ markedly in terms of levels in contrast to the previous data from Senegal where the higher concentrations have been reported for MSP3-b, MSP3-c and MSP3-d (6). This difference may be due to the malaria transmission level which is higher in our setting compared with Dielmo (Senegal), to the population genetic backgrounds and to our small sample size.

Cell-mediated immunity was investigated by measuring proliferative responses and IFN-γ measured by ELISA in proliferation culture supernatant. The SI for MSP3-LSP (12) and the four overlapping peptides measured before the first injection in October (day 0), just at the end of the peak of malaria high transmission period, was low and similar in both vaccine groups. The low SI may be due to the high background at this period of malaria transmission and this may be explained by the high antigenic pressure to which the study volunteers were exposed at the time of blood sampling. The T-cell response in individuals exposed to high antigenic pressure is uncertain when assessing the immune responses *in vitro*. During the high transmission season, the permanent exposure of the immune system to plasmodial antigens might induce increased levels of lymphocyte activation and hide the *in vitro* activity. This phenomenon was observed with high mean counts per minute of the negative control (data not shown). Proliferative responses and IFN-γ secretion in response to stimulation with MSP3-LSP (12) and the overlapping peptides were boosted following vaccination with MSP3-LSP. This immunogenicity is likely related to the four T-cell epitopes previously identified in malaria endemic area (6). The geometric means of the SI and the proportion of volunteers with high SI as well as the concentrations of IFN-γ in the supernatants were high in MSP3-LSP vaccinated volunteers compared with baseline (D0) and to the control group. Merozoite surface protein-3 long synthetic peptide appears to stimulate cell-mediated immunity even in these semi-immune volunteers with some pre-existing immunity as the result of many years of natural exposure. This finding is accordance with the phase 1a trial in naïve volunteers where MSP3-LSP proved able to trigger in humans a very high prevalence of strong T-cell responses, as shown by the high proliferation indices and IFN-γ production (11).

T-cell responses to the four peptides spanning the C-terminal part of MSP3 were generally lower than the responses to the full MSP3-LSP measured in parallel (12). The results indicate that these peptides have also been able to boost T-cell responses in MSP3-LSP vaccine group showing that the T-cell immunogenicity is likely related to these four T-cell epitopes previously identified in humans living in malaria endemic area (6). It also indicates that each of these four peptides tested defined at least one T-cell epitope and IFN-γ production results suggest that at least the responding cells belonged to the Th1-like type.

## Conclusion

Our data suggest that humoral responses to the MSP3 peptides were not boosted in semi-immune adults immunized with the full MSP3-LSP, perhaps because of high baseline antibody levels. However, there is some indication that the vaccine was able to illicit cellular immune responses in these semi-immune volunteers. Merozoite surface protein-3 long synthetic peptide is immunogenic in naïve adult volunteers and may therefore be more immunogenic in young children in endemic areas who have had less time to develop acquired immunity that the adults reported on here.
